# The Effect of Dietary Supplementation on Physical Performance in Adolescent Male Soccer Players Infected with SARS-CoV-2

**DOI:** 10.3390/nu17030527

**Published:** 2025-01-31

**Authors:** Andreea-Consuela Timnea-Florescu, Alexandru Dinulescu, Ana Prejmereanu, Olivia Carmen Timnea, Alexandra Floriana Nemes, Roxana Maria Nemes

**Affiliations:** 1 Faculty of Medicine, Titu Maiorescu University, 040441 Bucharest, Romania; andreea.timnea@doc.utm.ro (A.-C.T.-F.); roxanamarianemes@gmail.com (R.M.N.); 2 Chiajna Medical Center Dudu, 077041 Chiajna, Romania; 3 Faculty of Medicine, “Carol Davila” University of Medicine and Pharmacy, 050474 Bucharest, Romania; ana.prejmereanu@rez.umfcd.ro (A.P.); alexandra-floriana.nemes@drd.umfcd.ro (A.F.N.); 4 Departament of Pediatrics, Emergency Hospital for Children “Grigore Alexandrescu”, 011743 Bucharest, Romania; 5 Faculty of Physical Education, Sports and Physiotherapy, The Romanian-American University, 012101 Bucharest, Romania; 6 “Marius Nasta” Institute of Pneumophtisyiology, 050159 Bucharest, Romania

**Keywords:** COVID-19, SARS-CoV-2, adolescent athletes, nutrition intervention, dietary supplementation

## Abstract

Background/Objectives: The impact of the COVID-19 infection on athletes was reported to influence physical health, both decreasing performance and increasing the risk of injuries. This study aimed to assess the physical performance (maximal aerobic power, muscle function, and speed) of a group of male adolescent soccer players before and after COVID-19 infection and to compare the effects of nutrition intervention on physical performance. Methods: This study included 99 male soccer players, between 13 and 15 years old, that had mild SARS-CoV-2 infection. Their physical performance was evaluated in three periods (1 month before and 1 and 3 months after the infection). The subjects were divided into two groups, one with rigorous nutrition intervention and one without. Physical performance was evaluated through five tests: hand grip strength, 10 m sprint test, 30 m sprint test, beep test, and bench press. Results: A total of 20.2% had mild restrictions at spirometry after infection. One month after the infection, four of the five tests (hand grip strength, 10 m sprint test, 30 m sprint test, and beep test) showed statistically significantly (*p* < 0.005) better results in the nutrition intervention group. The same difference in results in the four tests was maintained 3 months after infection (*p* < 0.005). Conclusions: Nutrition intervention with a strict dietary plan and an increase in daily calories and protein and also vitamin and mineral supplements in young athletes may be effective for faster recovery of physical parameters from COVID-19 infection, and its beneficial effects should be studied further in this infection as well as in other respiratory tract infections.

## 1. Introduction

Coronavirus disease 19 (COVID-19), caused by infection with SARS-CoV-2, emerged first in December 2019 and later spread. In March 2020, the World Health Organization (WHO) declared it a pandemic. The clinical manifestations of this infection varied, with the most commonly reported being fever and respiratory symptoms [1,2,3,4]. The prevalence of the disease in adolescents ranges from 20% to 30%, with this age group being less infected than adults but more than small children [5,6,7]. The symptomatology of this infection in adolescents is generally mild, with an estimated prevalence of 80% asymptomatic and mild cases with good prognosis, the most common symptoms being fever, headache, hyposmia, cough, and sore throat. Almost 20% have been severe cases with severe respiratory failure and even deaths, predominantly among those with comorbidities such as obesity, asthma, epilepsy, and immunosuppression [4,8,9,10,11].

The impact of the COVID-19 infection on athletes was reported to influence both physical health, decreasing performance and increasing the risk of injuries in those who returned to the sport, and psychological health, with an increase in anxiety and other mental health problems. Aside from the disease, the lockdown influenced performance through lack of proper training, which increased body mass [12,13,14,15,16,17]. It is worth mentioning that athletes, however, reported lower levels of anxiety and post-traumatic stress than the general healthy population. This can be explained by the anxiolytic and antidepressant-like effects of exercise [12,17,18]. A study conducted in Romania that included athletes from different sports reported that the psychological impact was lower in football (soccer) than in other sports [19].

A well-balanced diet is needed for young athletes for proper growth and performance optimization. For sports practitioners at a professional level, nutrition should consist of multidisciplinary support from coaches, dietitians, and nutritionists. Supplements should be considered only under nutritional and medical control [20,21]. To maintain the health of athletes during the lockdown, dietary balance programs were implemented with adequate nutritional intakes of macronutrients and, particularly, micronutrients (vitamins and minerals) [17]. Physical tests are crucial for football players. Along with tactical preparation and mental status, they evaluate athletic performance. Physical preparation is also a method of injury prevention. Moreover, the correct physical preparation of an athlete is closely linked to achieving desired performance. Physical tests assess the player’s status, and based on them, the coach will know whether they can rely on the player in competition or even during training [22,23].

The objective of this study was to assess the physical performance in a group of male adolescent soccer players before (1 month) and for two periods of time after COVID-19 infection (1 month and 3 months) and to compare the influence of dietary supplements after the infection on the physical performance in two groups, one that received the supplementation with a strict dietary plan and one that did not receive supplementation.

## 2. Materials and Methods

This retrospective study included 99 male football (soccer) players between 13 and 15 years old with mild SARS-CoV-2 infection (2021–2022). The inclusion criteria for this study were professional male soccer players between the age of 13 and 15 years old, with clinical follow-up at Chiajna Medical Center, who had COVID-19. This study at first had 129 subjects, but 14 were lost to follow-up, 13 did not follow the diet, and 3 had injuries.

The pulmonary function was assessed through spirometry during the infection in all the subjects. Spirometry tests (MIR Spirolab IV; MIR—Medical International Research, Rome, Italy) were performed at the aforementioned clinic by a nurse supervised by a sports medicine specialist. Fitness and performance tests were performed periodically (weekly) by all participants: hand grip strength, 10 m sprint test, 30 m sprint test, beep test, and bench press (chest press). The tests were conducted at the training facilities of the team, both outdoors (football field) and in the gym. They were supervised by a physical trainer, physiotherapist, nurse, and doctor. To perform the tests, a dynamometer was needed for the hand grip test and a stopwatch, whistle, and cones for the 10 m sprint, 30 m sprint, and beep test, as well as a bench and a bar with different weights for the bench press. The performance of these subjects on this test was compared in 3 periods: 1 month before the SARS-CoV-2 infection, 1 month after the infection, and 3 months after the infection. The individuals were divided into 2 groups, 1 group that received dietary supplementation (*n* = 58) and another that did not (*n* = 41). The distribution was made based on the soccer clubs to which the players belonged, some clubs choosing to implement the dietary measures and others not.

The dietary plan they received was balanced and based on both energy requirements and the needs for carbohydrates, proteins, and healthy fats. This diet, along with vitamin supplementation, aimed to support both harmonious growth and athletic performance. The recommended caloric intake was between 2000 and 2500 kcal/day, divided into 3 main meals and 2 snacks, organized according to the time and type of training. In Table 1, there is an example of a meal schedule used, and in Table 2 and Table 3 are the recommended dietary supplements in the summer–autumn and winter–spring periods (the sports training periods). The nutritional objectives before competition were loading the body’s glycogen stores (carbohydrate loading), adequate hydration (fluid losses were corrected as follows: for every liter lost by sweat gradual rehydration, with 1.5 L of isotonic solution), avoiding gastrointestinal discomfort, adjusting food intake according to the time of day, and to modify the diet based on individual needs and preferences [24].

The players had 1 soccer game per week. T0 represents the day of the game. Recovery is the day after the game; then, the periods restart with the days before the next game, as such: T-5 (day 5 before the game), T-4 (day 4 before the game), T-3 (day 3 before the game), T-2 (day 2 before the game), and T-1 (day 1 before the game).

Physical fitness was compared between these 2 groups based on the results of the tests described before.

### Statistical Analysis

The data were collected in Microsoft Excel 2016, and the analysis was performed using IBM SPSS version 26. The Shapiro–Wilk test was used to analyze the quantitative variables’ distribution; the data were found to be non-normally distributed (Shapiro–Wilk, *p* ≤ 0.05), and they were reported as medians with interquartile ranges according to their distribution. Quantitative variables were tested between the independent groups using the Mann–Whitney U test and Independent Samples Test. Fisher’s exact test was used to determine (119) the nonrandom associations between categorical variables, with the Bonferroni method used (120) for correction. A minimal sample size was measured using the GPower 3.1.9.7 software. Based on the proposed assumptions of this study, the majority of the hypotheses were tested using comparisons of quantitative variables with non-parametric distribution between the two investigated groups using Mann–Whitney U tests. Also, considering the design of this study having two treatment groups separated by the existence of the administration of treatment, the dimensions of the effect sizes for the investigated parameters would be high (based on the probable influence of the treatment). As such, considering a high size effect—d = 0.8, with a level of significance of 0.05 and a minimal statistical power of 0.8—the minimal sample size for the proposed study (considering a minimal asymptotic relative efficiency—min A.R.E., of the Mann–Whitney U test) would be *n* = 60, with an allocation ratio of 1 between groups. Also, in the case of the usage of an optimal statistical power of 0.9, the minimal sample size would be *n* = 80. Considering that the final sample size used in this study was *n* = 91 (*n* = 41 in the first group and *n* = 58 in the second group), based on the criteria mentioned above, this study can be considered valid.

## 3. Results

Most subjects were 13 years old (49.5%), and the 14- and 15-year-olds were equally represented (25.3%). In 20 (20.2%) of them, the spirometry was modified, showing mild restriction. The rest 79 (79.8%) had normal pulmonary function. In total, 58 (58.5%) subjects received dietary supplementation, and 41 did not.

Table 4 represents the characteristics of the two groups. There were no statistically significant differences between the ages, the BMIs, or the percentages of mild restrictions on spirometry in the two groups (*p* > 0.05).

### 3.1. Hand Grip Strength

The median value of the grip strength in both groups was the lowest 1 month after SARS-CoV-2 infection (19.5 (17.8–26.5)) and was higher 3 months after the infection (24.8 (20.1–28.5)) than 1 month before (24.2 (20.2–27.9)) (Table 5).

There was no difference between the results of the hand grip strength in the two groups before the SARS-CoV-2 infection (*p* = 0.206), but 1 month and 3 months after the infection, the results of the grip strength were better in the dietary supplementation group: 26.5 (18.5–26.9) vs. 18.1 (17.2–24) 1 month after (*p* < 0.05) and 27.3 (23.4–29.3) vs. 21.2 (18.4–25.6) 3 months after (*p* < 0.05) (Table 6).

### 3.2. 10 m Sprint Test

The median value of the 10 m sprint test in both groups was higher 1 month after SARS-CoV-2 infection (2.2 (2.1–2.4)) than 1 month before and 3 months after the infection, and the values were better 3 months (2.1 (2.1–2.3)) after than 1 month before the COVID-19 (2.2 (2.1–2.3)) (Table 7).

There was no difference between the results of the 10 m sprint test in the two groups before the SARS-CoV-2 infection (*p* = 0.164), but 1 month and 3 months after the infection, the results were better in the dietary supplementation group: 2.2 (2.1–2.3) vs. 2.3 (2.2–2.6) 1 month after (*p* = 0.001) and 2.1 (2.1–2.2) vs. 2.2 (2.1–2.5) 3 months after (*p* = 0.002) (Table 8).

### 3.3. 30 m Sprint Test

The median value of the 30 m sprint test in both groups was higher 1 month before SARS-CoV-2 infection (5.2 (5–5.5)) than 1 month and 3 months after the infection, and the values were better 3 months after (5.1 (4.9–5.4)) than 1 month after (5.2 (5–5.4)) the COVID-19 (Table 9).

There was no difference between the results of the 30 m sprint test of the two groups before the SARS-CoV-2 infection (*p* = 0.091), but 1 month and 3 months after the infection, the results were better in the dietary supplementation group: 5.1 (5–5.3) vs. 5.4 (5.2–5.5) 1 month after (*p* < 0.001) and 4.9 (4.9–5.3) vs. 5.2 (5–5.4) 3 months after (*p* = 0.001) (Table 10).

### 3.4. Beep Test

The median values of the beep test in both groups were the same one month before and one month after the SARS-CoV-2 infection (7.5 (7–8)), but the value was higher three months after the infection: 8 (7–8.5) (Table 11).

There was no difference between the results of the beep test in the two groups before the SARS-CoV-2 infection (*p* = 0.835), but 1 month and 3 months after the infection, the results were better in the dietary supplementation group: 7.5 (7.5–8) vs. 7 (7–7.5) 1 month after (*p* < 0.001) and 8 (7–8.6) vs. 7.5 (7–8) 3 months after (*p* < 0.001) (Table 12).

### 3.5. Bench Press

The median bench press values in both groups were the same one month before and one month after the SARS-CoV-2 infection (20 (15–25)), but the value was higher three months after the infection (25 (20–30)) (Table 13).

There was no difference between the results of the bench press in the two groups in all three periods of time in the Mann–Whitney U test (*p* > 0.05).

## 4. Discussion

The aim of this study was to report the physical performance (maximal aerobic power, muscle function, and speed) and recovery in a cohort of adolescent soccer players that had COVID-19 infection. The performance was compared before (1 month) the infection and after (1 month and 3 months) the infection, and also, there was a comparison between the recovery and the performance of two groups: one that received dietary supplementation (the diet is discussed in details in the methods section) and one group that did not receive supplementation.

We reported mild restriction (defined as 70–80% predicted) at spirometry in 20.2% (20/99) individuals included in this study; no other respiratory alteration was registered [25]. These findings are consistent with the literature, where it is reported that 80–90% of COVID-19 patients are asymptomatic or have mild symptoms [26]. Also, the restrictive pattern at spirometry is predominantly described in COVID-19 infection, but there is not a consensus about the duration of these modifications after the infection. One study published by Tamminen et al. (2022) that included 43 patients described resolution of the lung function after 2 months; Fumagalli et al. (2021) reported improvement of the pulmonary function after 6 weeks in a case series of 13 patients, but still lower than the upper limit of normal. Abbas et al. (2024) showed in a prospective study that included 198 patients that 33.5% of those subjects had still restrictive respiratory patterns 6–8 months after recovery from the infection [27,28,29,30,31].

To assess the physical performance of the young soccer players included in this study, five physical tests were used: Hand grip strength is a test, used frequently in the sporting populations, that is used to assess muscle function and overall sport performance [32,33]. It was demonstrated by James et al. (2017) to have a correlation in young soccer players [34]. The sprint tests (10 m and 30 m) are valid physical tests used in soccer, with Zhang et al. (2022) even describing a correlation between the 10 m test and the change-in-direction ability of the soccer players [35,36]. The 20 m multistage fitness test (beep test) determines maximal aerobic power [37,38]. The bench press is used to stimulate strength growth in the upper body, and adding it to soccer training is beneficial for overall physical capacity development [39,40].

In four out of the five tests (except the bench press) that evaluated physical performance, the results 1 month after the COVID-19 infection were poorer than the results 1 month before the infection, being in consensus with the literature, where multiple studies describe an increase in fatigue and a decrease in physical performance in the general population and athletes [12,13,14,15,16,40,41,42]. Kim et al. (2023) reported that 61.5% of 226 elite university athletes experienced difficulties in ordinary training after the infection. Most described it as a lack of energy or easy fatiguability [13]. In all five tests, the performance at 3 months after the infection was better than 1 month after the infection, showing recovery in the fitness of the athletes, in consensus with Schroeder et al. (2023), who reported that in 104 adult triathletes, there was a return to normal fitness of only 39% in less than a month, but Vollrath et al. (2022) published a study with 60 adult athletes included in the COVID-19 in German Competitive Sports (CoSmo-S) study, which reported that 61.7% had persistent symptoms even at the 3-month postinfection examination, and a 3-month follow-up study published by Clavario et al. (2021) with 200 adult patients (which were not athletes) discovered that almost 1/3 of them had functional limitations [14,42,43].

The beneficial effects of dietary supplementation on SARS-CoV-2 infection were studied in the general population at the beginning of the pandemic [44,45,46,47]. The European Society for Clinical Nutrition and Metabolism (ESPEN) recommended that nutritional support with omega-3 polyunsaturated fatty acids (PUFAs), iron, zinc, vitamin D, and vitamin B should be considered in patients with COVID-19 infection. Even those without a risk of malnutrition should receive adequate intake of nutrients, principally 25–30 kcal/day and 1.5 g protein/day [43,46,47]. Pavlidou et al. (2024) published a review on this subject that included 26 studies in which they concluded that dietary supplementation with macronutrients and micronutrients shows promise both before and during COVID-19 infection [46]. Another review published by Younes (2024) reports a beneficial effect of calcium, magnesium, iron, and vitamin D supplementation [45]. Nutrition intervention (dietary supplementation, carbohydrate ingestion, and diet plans) is used in professional soccer and other sports to enhance physical abilities, and the use of supplements has already spread from professional athletes to the general population, with known beneficial effects of the antioxidant, creatine, and adequate daily protein intake [48,49,50,51,52,53,54]. The International Olympic Committee (IOC) recognizes some substances like sodium bicarbonate, creatine, nitrate, and beta-alanine as beneficial for athletes [55].

The recommended diet is a high-calorie diet that follows the principles of a healthy and balanced diet for athletes. The variety of macronutrients (proteins, healthy fats, and carbohydrates), along with micronutrients, plays a fundamental role in supporting both physiological processes and energy production, particularly by maintaining metabolic balance. Additionally, it influences immune function and muscle growth [56,57]. Proteins are made up of amino acids that are vital for tissue repair, immune function, enzyme activity, and muscle growth. The amino acids in these foods contribute to protein synthesis in muscles and other tissues. Additionally, certain amino acids, such as glutamine and alanine, are important for metabolic processes in the liver. It is recommended to consume adequate amounts of protein, typically between 1.2 and 2 g of protein per kilogram of body weight, depending on the intensity of training and the overall condition of the body [58,59]. Carbohydrates are an important source of energy, especially during the recovery period, when the body needs resources to combat the effects of viruses and to support lightly adapted physical activities. Complex carbohydrates, such as whole grains, brown rice, sweet potatoes, and vegetables, are good options. After physical activity, carbohydrates serve as the primary source of energy for both the brain and muscles. They also play an essential role during physical activity. Glucose is the final product of carbohydrate breakdown and represents the fastest available energy source. Carbohydrate loading before games was found to be effective in soccer players [60,61]. Fats play an important role in the structure of the cell membrane, facilitating the absorption of fat-soluble vitamins. They also represent a rich source of energy. Fats are important for the optimal functioning of the nervous system and for reducing inflammation. Good sources of fats include avocados, vegetable oils (olive, avocado), nuts, and fatty fish (salmon, sardines) [62]. Vitamins and minerals play an important role along with the diet in supporting athletic performance. Together, these support the body’s functions, including metabolic, immune, hormonal, and enzymatic processes. Consideration should be given to competition periods, seasons, and the type of training on each particular day. Minerals, antioxidants, and electrolytes have been recommended to support athletic performance and liver regeneration [57].

In this study, 58 (58.5%) received nutrition intervention after infection, with a dietary plan that provided an increase in daily calories and protein and also micronutrients (vitamins and minerals) and dietary supplements (as was described in the Methods section), and 41 (41.5%) did not. After the dietary supplementation, the physical performance was compared using the five tests described before (hand grip strength, 10 and 30 m sprint test, beep test, and bench press). One month after the infection, four of the five tests (excluding the bench press) showed statistically significantly better results in the nutrition intervention group: hand grip strength test: 26.5 (18.5–26.9) vs. 18.1 (17.2–24) (*p* < 0.05); 10 m sprint test: 2.2 (2.1–2.3) vs. 2.3 (2.2–2.6) (*p* = 0.001); 30 m sprint test: 5.1 (5–5.3) vs. 5.4 (5.2–5.5) (*p* < 0.001); and beep test: 7.5 (7.5–8) vs. 7 (7–7.5) (*p* < 0.001). The same difference in results in the four tests was also maintained 3 months after the infection, with hand grip strength test: 27.3 (23.4–29.3) vs. 21.2 (18.4–25.6) (*p* < 0.05); 10 m sprint test: 2.1 (2.1–2.2) vs. 2.2 (2.1–2.5) (*p* = 0.002); 30 m sprint test: 4.9 (4.9–5.3) vs. 5.2 (5–5.4) (*p* = 0.001); and beep test: 8 (7–8.6) vs. 7.5 (7–8) (*p* < 0.001), concluding that the nutrition intervention had a significant beneficial effect on the physical performance 1 month and 3 months after the infection. Grant et al. published a review in 2020 that advocated using vitamin D3 supplementation on athletes during the COVID-19 pandemic. Śliż et al. (2022) reported the effect of nutrition on physical performance in 49 adult endurance athletes. Unsaturated fatty acid consumption was linked with better fat-free mass (*p* = 0.031), and the addition of salt increased maximal speed/power (*p* = 0.024) [63]. Aside from COVID-19 infection, Łagowska and Bajerska published a meta-analysis in 2021 of 14 randomized controlled trials that provided evidence for probiotic supplementation among professional athletes as an effective way to decrease symptom severity scores in upper respiratory tract infections [64].

Limitations: As described in the introduction section, COVID-19 infection and the lockdown had a significant impact on psychological health, an impact that was not measured in this study. Other influencing factors, such as baseline fitness levels, training habits, mental health status, and socio-economic background, were not controlled. The physical tests do not comprehensively represent all aspects of athletic performance. This study focuses solely on male adolescent soccer players; therefore, the findings may not be generalizable to other populations.

## 5. Conclusions

Mild restriction was reported in the spirometry in approximately 1/5 of the cases. The physical tests showed a decrease in performance 1 month after the COVID-19 infection but with a recovery 3 months after. The group with the dietary supplementation had better results in four of the five physical tests. Nutrition intervention with a strict dietary plan and increases in daily calories and protein and also vitamin and mineral supplements in young athletes may be effective for a faster recovery of the physical parameters from COVID-19 infection, and its beneficial effects should be studied further in this infection as well as in other respiratory tract infections.

## Figures and Tables

**Table 1:** 1 day menu.

Meals	
Breakfast	Cereal (50 g) with milk (100 mL), omelet (2 eggs or 3 egg whites) with ham (50 g) + vegetables (unlimited) + 2 slices of bread (25 g) + butter (10 g) + honey (1 small pack, 15 g) or croissant with chocolate(50 g) + some fresh fruits (100 g: 1 banana or 2 oranges or 3 kiwis)—500 kcal
Lunch	Pasta salad (50 g, uncooked) with tuna (50 g) + frittata with tomatoes (50–100 g) and ricotta (50 g) + 4 slices of bread (50 g) + cookies with no cream (50 g) or grapes (100 g) or bananas (1 banana)—750 kcal
Dinner	Vegetable cream soup (200 mL) + chicken skewers (200 g) with vegetables (unlimited) + baked potatoes (1 big, 2 small) 100 g + chocolate cake (120 g)—800 kcal
Snack 1	1 banana (100 g) + mix of dried fruits and seeds (30 g)
Snack 2	Chocolate cereal bar or oatmeal (75 g) + Greek yogurt with 10% fat (100 g)—600 kcal

**Table 2:** Recommended dietary supplement model during the summer–autumn competition period.

	Recovery	T-5	T-4	T-3	T-2	T-1	T0 (Game Day)
Morning	Tonotil *, 1 vial per day	Vitamins A-Z **, 1 tablet per day	Vitamins A-Z, 1 per day	Vitamins A-Z, 1 tablet per day	Tonotil, 1 vial per day	Tonotil, 1 vial per day	Tonotil, 1 vial per day
Lunch	Vitamin C, 1000 mg; 1 tablet per day				Vitamins A-Z, 1 tablet per day	MaxiMag ***, 1 pill per day	Carcel Stop ****, 1 tablet per day
Dinner	Liv52 ^5*^, 1 tablet per day	Liv52, 1 tablet per day	Liv52, 1 tablet per day	Liv52, 1 tablet per day	Liv52, 1 tablet per day	Sargenor ^6*^, 1 vial per day	Sargenor, 1 vial per day

* Tonotil vial: L-phosphoserine—60 mg; L-glutamine—75 mg; L-phosphothreonine—20 mg; arginine hydrochloride—150 mg; vitamin B12—0.5 mg. ** Vitamin A-Z tablet: vitamin A—400 µg, lutein—1500 µg, vitamin D—5 µg, vitamin E—10 mg, vitamin K—20 µg, vitamin C—150 mg, vitamin B1—3.5 mg, vitamin B2—4 mg, niacin—18 mg, vitamin B6—5 mg, folic acid—450 µg, vitamin B12—2.5 µg, biotin—300 µg, pantothenic acid—12 mg, calcium—137 mg, phosphorus—105 mg, magnesium—56.3 mg, iron—2.1 mg, zinc—5 mg, copper—900 µg, selenium—10 µg, chromium—25 µg, molybdenum—20 µg. *** MaxiMag pill: ionic magnesium—375 mg, vitamin B6—1.4 mg. **** Carcel stop tablet: magnesium—300 mg, chromium—30 µg, potassium—300 mg, zinc—5 mg, iron—3.5 mg, vitamin B6—4.2 mg, vitamin B12—5 µg. ^5*^ Liv 52: capparis spinosa—65 mg, cichorium intybus—65 mg, mandur bhasma—33 mg, solanum nigrum—32 mg, terminalia arjuna—32 mg, cassia occidentalis—16 mg, achillea millefolium—16 mg, tamarix gallica—16 mg. ^6*^: Sargenor: arginine aspartate—950 mg, vitamin B6—4 mg, biotin—150 mcg, magnesium—83.3 mg.

**Table 3:** Recommended dietary supplements model during the winter–spring competition period.

	Recovery	T-5	T-4	T-3	T-2	T-1	T0 (Game Day)
Morning	Tonotil, 1 vial per day Vitamin D, 1000 UI; 1 tablet per day Vitamin C, 1000 mg; 1 tablet per day	Vitamin C, 1000 mg; 1 tablet per day Vitamin D, 1000 UI; 1 tablet per day	Supradyn, 1 per day Vitamin D, 1000 UI; 1 tablet per day	Supradyn, 1 per day Vitamin D, 1000 UI; 1 tablet per day	Tonotil, 1 vial per day Vitamin D, 1000 UI; 1 tablet per day	Tonotil, 1 vial per day Vitamin D, 1000 UI; 1 tablet per day	Tonotil, 1 vial per day Vitamin D, 1000 UI; 1 tablet per day
Lunch							Vitamin A-Z, 1 tablet per day
Dinner	Silimarina Forte *, 1000 mg; 1 tablet per day	Silimarina Forte *, 1000 mg; 1 tablet per day	Silimarina Forte *, 1000 mg; 1 tablet per day	Silimarina Forte *, 1000 mg; 1 tablet per day	Silimarina Forte *, 1000 mg; 1 tablet per day	Sargenor, 1 vial per day	Sargenor, 1 vial per day

* Silimarina forte: extract of silybum marianum.

**Table 4:** Characteristics of the two groups.

Group	No Dietary Supplementation Group (N-41)	Dietary Supplementation Group (N-58)	*p*
Age (years)	14 (13–14)	14 (13–15)	0.398 *
BMI (kg/m^2^)	18.2 ± 2.3	18.5 ± 1.5	0.481 **
Spirometry with mild restrictions (number/percent)	8 (19.5%)	12 (20.6%)	0.732 ***

* Mann–Whitney U test. ** Independent Samples Test. *** Fisher’s exact test.

**Table 5:** Hand grip strength median values by SARS-CoV-2 infection time.

Time of Examination by SARS-CoV-2 Infection	Grip Strength (kg) Median (IQR)
1 month before (no diet in both groups)	24.2 (20.2–27.9)
1 month after	19.5 (17.8–26.5)
3 months after	24.8 (20.1–28.5)

**Table 6:** Grip strength median values by SARS-CoV-2 infection time in the two groups.

	Hand Grip Strength (kg) Median (IQR)		
Time of Examination by SARS-CoV-2 Infection	No Dietary Supplementation Group	Dietary Supplementation Group	*p* *
1 month before (no diet in both groups)	22.3 (20.5–26.3)	25.3 (20–28.3)	0.206
1 month after	18.1 (17.2–24)	26.5 (18.5–26.9)	**<0.05**
3 months after	21.2 (18.4–25.6)	27.3 (23.4–29.3)	**<0.05**

* Mann–Whitney U test.

**Table 7:** The 10 m sprint test median values by SARS-CoV-2 infection time.

Time of Examination by SARS-CoV-2 Infection	10 m Sprint Test (s) Median (IQR)
1 month before (no diet in both groups)	2.2 (2.1–2.3)
1 month after	2.2 (2.1–2.4)
3 months after	2.1 (2.1–2.3)

**Table 8:** The 10 m sprint test median values by SARS-CoV-2 infection time in the two groups.

10 m Sprint Test (s) Median (IQR)			
Time of Examination by SARS-CoV-2 Infection	No Dietary Supplementation Group	Dietary Supplementation Group	*p* *
1 month before (no diet in both groups)	2.2 (2.1–2.2)	2.2 (2.1–2.3)	0.164
1 month after	2.3 (2.2–2.6)	2.2 (2.1–2.3)	**=0.001**
3 months after	2.2 (2.1–2.5)	2.1 (2.1–2.2)	**=0.002**

* Mann–Whitney U test.

**Table 9:** The 30 m sprint test median values by SARS-CoV-2 infection time.

Time of Examination by SARS-CoV-2 Infection	30 m Sprint Test (s) Median (IQR)
1 month before (no diet in both groups)	5.2 (5–5.5)
1 month after	5.2 (5–5.4)
3 months after	5.1 (4.9–5.4)

**Table 10:** The 30 m sprint test median values by SARS-CoV-2 infection time in the two groups.

30 m Sprint Test (s) Median (IQR)			
Time of Examination by SARS-CoV-2 Infection	No Dietary Supplementation Group	Dietary Supplementation Group	*p* *
1 month before (no diet in both groups)	5.3 (5.2–5.5)	5.1 (5–5.5)	0.091
1 month after	5.4 (5.2–5.5)	5.1 (5–5.3)	**<0.001**
3 months after	5.2 (5–5.4)	4.9 (4.9–5.3)	**=0.001**

* Mann–Whitney U test.

**Table 11:** Beep test median values by SARS-CoV-2 infection time.

Time of Examination by SARS-CoV-2 Infection	Beep Test (Levels) Median (IQR)
1 month before (no diet in both groups)	7.5 (7–8)
1 month after	7.5 (7–8)
3 months after	8 (7–8.5)

**Table 12:** Beep test median values by SARS-CoV-2 infection time in the two groups.

Beep Test (Levels) Median (IQR)			
Time of Examination by SARS-CoV-2 Infection	No Dietary Supplementation Group	Dietary Supplementation Group	*p* *
1 month before (no diet in both groups)	7.5 (7–8)	7.5 (6.5–8)	0.835
1 month after	7 (7–7.5)	7.5 (7.5–8)	**<0.001**
3 months after	7.5 (7–8)	8 (7–8.6)	**<0.001**

* Mann–Whitney U test.

**Table 13:** Bench press median values by SARS-CoV-2 infection time.

Time of Examination by SARS-CoV-2 Infection	Bench Press (kg) Median (IQR)
1 month before (no diet in both groups)	20 (15–25)
1 month after	20 (15–25)
3 months after	25 (20–30)

## Data Availability

The data can be shared up on request.
